# Advancing complexity science in healthcare research: the logic of logic models

**DOI:** 10.1186/s12874-019-0701-4

**Published:** 2019-03-12

**Authors:** Thomas Mills, Rebecca Lawton, Laura Sheard

**Affiliations:** Bradford Institute for Health Research, Temple Bank House, Bradford Royal Infirmary, Duckworth Lane, Bradford, BD9 6RJ UK

**Keywords:** Logic models, Program theory, Implementation models, Complexity, Complexity science, Complex interventions, Facilitation, Context

## Abstract

**Background:**

Logic models are commonly used in evaluations to represent the causal processes through which interventions produce outcomes, yet significant debate is currently taking place over whether they can describe complex interventions which adapt to context. This paper assesses the logic models used in healthcare research from a complexity perspective. A typology of existing logic models is proposed, as well as a formal methodology for deriving more flexible and dynamic logic models.

**Analysis:**

Various logic model types were tested as part of an evaluation of a complex Patient Experience Toolkit (PET) intervention, developed and implemented through action research across six hospital wards/departments in the English NHS. Three dominant types of logic model were identified, each with certain strengths but ultimately unable to accurately capture the dynamics of PET. Hence, a fourth logic model type was developed to express how success hinges on the adaption of PET to its delivery settings. Aspects of the Promoting Action on Research Implementation in Health Services (PARIHS) model were incorporated into a traditional logic model structure to create a dynamic “type 4” logic model that can accommodate complex interventions taking on a different form in different settings.

**Conclusion:**

Logic models can be used to model complex interventions that adapt to context but more flexible and dynamic models are required. An implication of this is that how logic models are used in healthcare research may have to change. Using logic models to forge consensus among stakeholders and/or provide precise guidance across different settings will be inappropriate in the case of complex interventions that adapt to context. Instead, logic models for complex interventions may be targeted at facilitators to enable them to prospectively assess the settings they will be working in and to develop context-sensitive facilitation strategies. Researchers should be clear as to why they are using a logic model and experiment with different models to ensure they have the correct type.

**Electronic supplementary material:**

The online version of this article (10.1186/s12874-019-0701-4) contains supplementary material, which is available to authorized users.

## Background

The case for process evaluations is now well-established in healthcare research following publication of the Medical Research Council (MRC) guidance in 2008 [1]. The MRC guidance advocated for the greater use of qualitative, process evaluations to produce theory of how interventions work (sometimes referred to as “programme theory” or “theory of change”), said to be necessary to ensure their optimal development and use [1]. Yet, questions are increasingly being asked of whether the MRC guidance does enough to address the challenges involved in evaluating complex interventions [2–6]. Scholars influenced by complexity science have argued that the MRC guidance is appropriate only for *complicated* interventions that work roughly the same way in different settings. *Complex* interventions, by contrast, seek to change social systems such that pre-existing contextual factors shape the form that they take [2–6]. Feedback loops provide the opportunity for those delivering and receiving the intervention to adapt it to context, potentially changing the activities to be delivered and the outcomes that are produced [5]. An example of this dynamic can be found in public health in the case of school-based nutrition education interventions. A qualitative exploration of their work found that nutritionists’ practices varied according to their past experiences and each school setting and they strategically adapted interventions to keep people engaged, exhibiting an intuitive awareness of the needs and goals of students and teachers. This implies a blurring of the boundaries between interventions and context that is difficult to reconcile with traditional evaluation techniques [7].

Significant debate has taken place about the methods suitable for designing and evaluating these more complex interventions. Greater focus on the developmental stage of interventions is said to be necessary and formative methods that allow interventions to adapt on implementation are increasingly advocated [4, 5]. Yet, while the need for theoretical evaluation of complex interventions continues to be recognised, the role of logic models in this new research paradigm is unclear.

Logic models are assigned the role, in process evaluations, of representing the underlying theory of interventions in simple, diagrammatical form (see Additional file 1: Appendix 1 for a glossary of key terms related to logic models). For their advocates, they can be useful to help evaluators develop understanding of exactly how interventions produce outcomes [1, 3], to organise empirical data and specify process and outcome measures for the purposes of evaluation [8] and/or to provide a talking point for stakeholders to forge consensus on the need for change and how to go about it [9]. Logic models can also be useful to demonstrate programme logics to funders and aid the process of knowledge transfer whereby research findings are applied outside of initial test sites [10]. Yet, existing guidance on logic modelling in healthcare research pays very little attention to the interaction between interventions and context [2–6]. Some have concluded that logic models have reached the limits of their use [4, 8, 10–16].

The utility of logic models has been a frequent topic of BMC Medical Research Methodology [8, 11, 17, 18]. Addressing the aforementioned debate, Greenwood et al. question whether logic models can represent the dynamics of complex interventions that adapt to context, stating that “no matter how sophisticated, a logic model alone is not sufficient, as complexity cannot be understood purely through qualitative description” [11]. We feel this is too quick a rejection of qualitative logic models. While logic model types that are currently dominant in healthcare research may be inadequate for describing complex, adaptive interventions, more flexible and dynamic types are possible. We demonstrate this with reference to our experience of developing and evaluating a Patient Experience Toolkit intervention. A typology of logic model types is proposed based on a scoping review of the literature, along with a formal methodology for developing dynamic models, referred to as “type 4” logic models. We hope this will help researchers to a) know which logic model type to use when evaluating interventions and b) overcome the challenges of modelling complex interventions.

## Main

### Modelling a patient experience improvement toolkit intervention

Various logic models were tested as part of an evaluation of a Patient Experience Toolkit (PET), developed to guide healthcare professionals through a facilitated process of reflecting and acting on patient experience data^1^. This process includes stages for setting up a multidisciplinary team, reflecting on patient feedback and making changes using QI techniques. Six hospital wards across three NHS Trusts in the North of England were involved in the study, specifically chosen to present very different contexts for the PET intervention. Ward teams and patient representatives worked with researchers in an action research project to implement and refine PET over the course of a year. The task of the evaluation was to develop generalisable theory of how the intervention works as a whole, using a logic model approach.

**Fig. 1:**
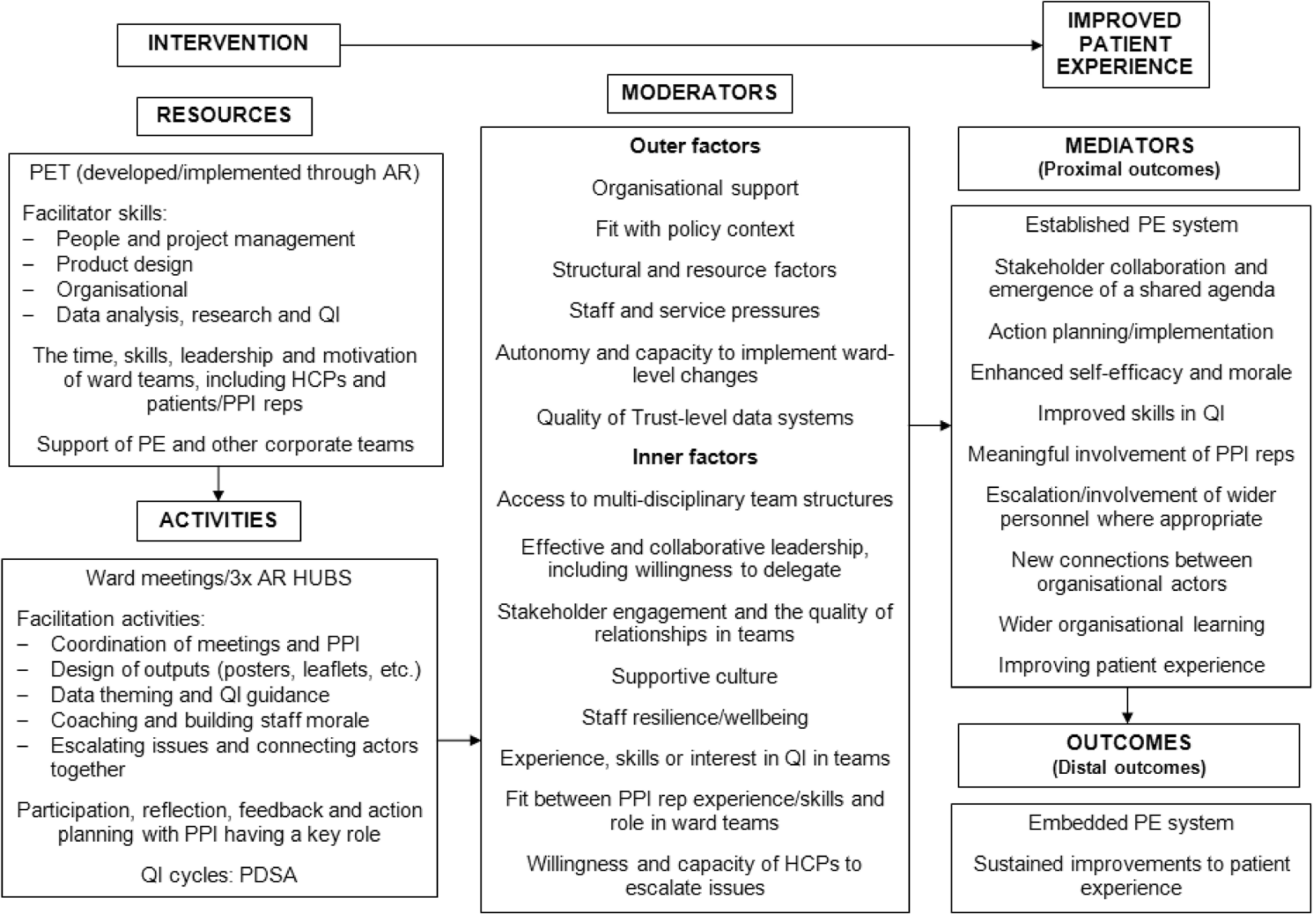
Initial Logic Model for the PET Intervention. The initial logic model focuses on the core levers of the intervention. An early finding of the evaluation was that the PET document was insignificant relative to the facilitation provided by the action researchers. Hence, facilitation skills are included as a key resource and the facilitation activities that supported PET’s delivery are listed in the activities section. The people involved in the study besides the action researchers, including HCPs, patients/PPI reps and PE/corporate teams, are also listed as an intervention resource as they were frequently identified as having contributed to outcomes. In addition, various mechanisms are included in the model that could be seen to be operating through the intervention, notably participation, reflection, feedback and action planning/QI cycles. The factors that moderated PET’s delivery, identified as either constraints on wards that struggled with implementation or enablers for achieving full implementation, are listed in the moderators section. Finally, while the ideal, distal outcome of a fully embedded patient experience system (with sustained improvements to patient experience occurring) was not achieved on any ward, the various proximal outcomes listed in the logic model could be identified across the participating wards

Figure 1 presents a logic model for the PET intervention. This was developed iteratively through an analysis of a large, qualitative dataset collected over the course of the project, using the framework method [19]. Logic model categories (intervention resources and activities, moderators and outcomes) informed the columns of the framework matrix and each ward were assigned a separate row, enabling the vast dataset to be organised, summarised and analysed in a way that was relevant to the logic model.

While the initial logic model structure proved useful as an organising framework for developing theory of the intervention, from the halfway point onwards TM was increasingly concerned that it was failing to accurately capture its underlying logics. Analysis of the ward columns in the framework matrix revealed significant divergences in the form the intervention was taking on, under the influence of the action researchers’ facilitation. The logic model, developed for all wards combined, was failing to capture the intervention’s dynamics in four main ways:

1. *Roles* – The roles and responsibilities of ward team members differed in accordance with their willingness and capacity to engage, with the action researchers adapting their role to fit each team. They carried out some of the facilitation tasks for one team which a ward manager or patient representative had done for another team.
2. *Interaction between the facilitation and moderators* – The action researchers could also be seen responding to the presence of moderators existing in each ward setting. For example, coaching was particularly prominent when ward cultures were perceived to be unsupportive of improvement work, characterised by low staff engagement, wellbeing and self-efficacy. Low organisational support and a lack of escalation channels could also be overcome by the action researchers establishing relationships with corporate staff. The initial logic model does not model this dynamism between the facilitation and moderating factors, implying they were experienced only as enablers or barriers.
3. *Irregular patterns of proximal outcomes* – Some of the proximal outcomes identified in the logic model, such as the emergence of a shared agenda, action planning/implementation and meaningful involvement of patient representatives, were apparent on all wards and can therefore be considered core “mediators” of PET. Yet, other proximal outcomes were linked to the action researchers’ efforts to overcome moderators that were specific to particular ward settings, such as improved ward culture or connections between actors.
4. *Proximal outcomes influencing later success* – Finally, the initial logic model does not show how the emergence of the proximal outcomes could strengthen the work of the project. Initial improvements and the emergence of proximal outcomes could create a more receptive context for the intervention, making later improvement efforts easier to implement.

### A typology of logic models used in healthcare research

**Fig. 2:**
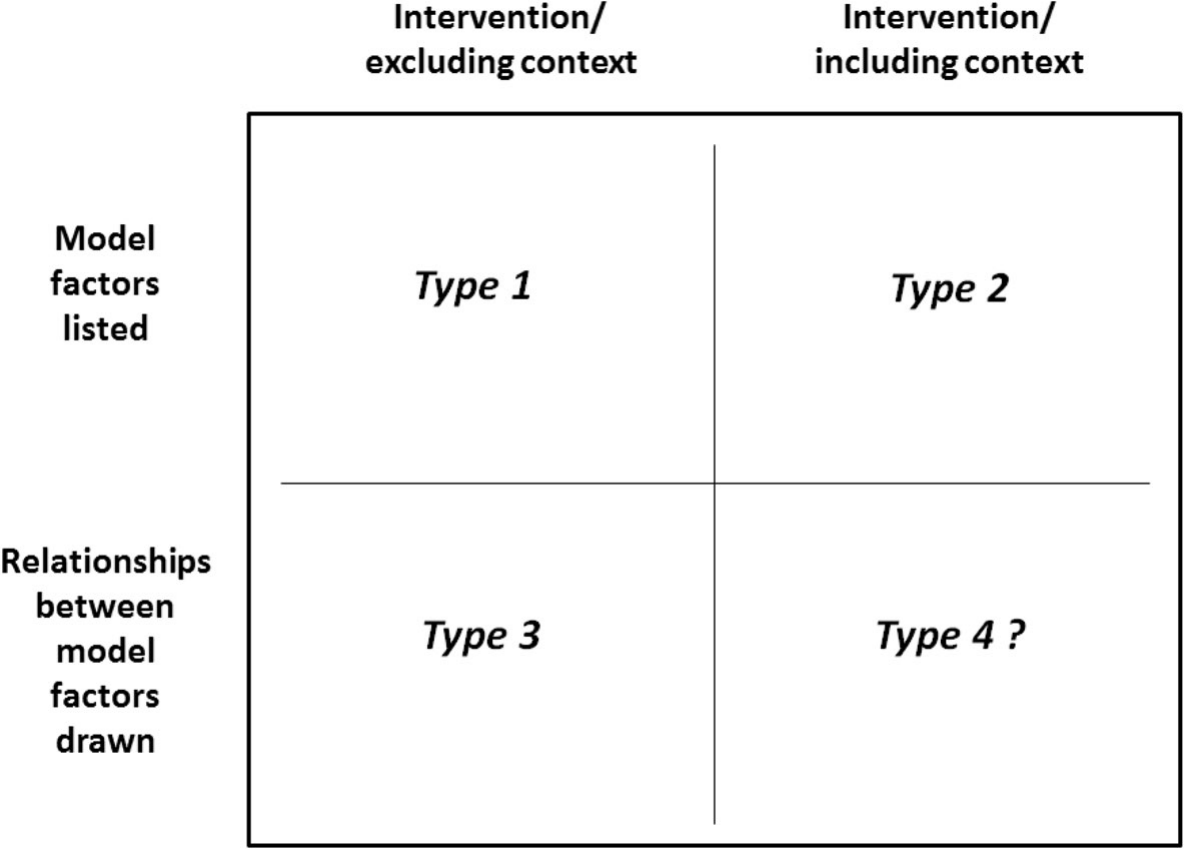
A Typology of Logic Models in Healthcare Research. Figure 2 describes logic models as having two key characteristics: firstly, whether they list model factors only or whether they also express the relationships between the factors; and, secondly, whether context is included as part of the model or whether it is omitted. While this typology implies the possibility of four types, most logic models take the form of one of three types. Logic models that model the dynamic interaction between interventions and context, the most appropriate type for complex interventions, are rare, hence the question-mark after type 4

The failure of the initial logic model to accurately describe the PET intervention led TM to assess the logic model field to see whether alternative approaches existed. A scoping review was carried out, using techniques derived from established guidance [20]. Academic databases (Medline/PubMed and ASSIA) and Google Scholar were used to identify relevant articles within both grey and published literature. Articles were included if they had a focus on health and either advocated for a particular approach to logic modelling or reviewed the field. Logic models were assessed in terms of their core characteristics and how they modelled complexity (i.e. as a factor of interventions or context). A typology was developed to reflect differences in this regard and this was refined over the course of the search. As the typology was being refined, papers were excluded if they did not offer any unique insight into a logic modelling approach. Nine key papers were identified as either offering a unique logic modelling approach [3, 21–24] or a review of the field which illuminated differences within logic modelling [3, 25–28]. Further analysis of these papers informed the construction of a four-pronged typology (see Fig. 2), after which TM assessed each type to see whether it could describe the PET intervention.

### Type 1 and type 2 logic models

**Fig. 3:**
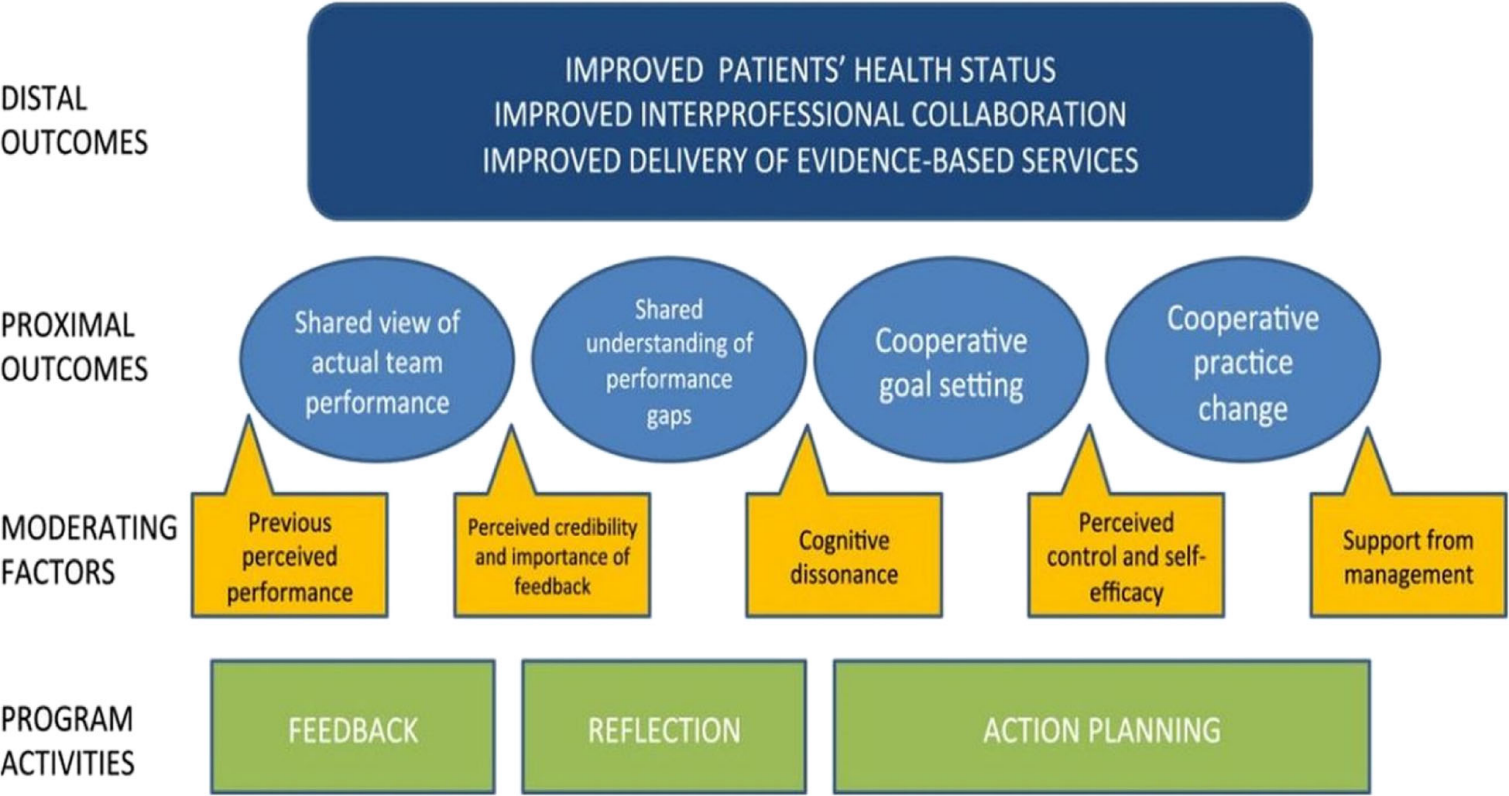
Example of a Type 2 logic model. *Source: Davidoff* et al.*, 2015* [23]*.* Some type 2 logic models account for complexity by moving to a higher degree of abstraction, listing intervention mechanisms instead of a precise list of intervention resources and activities (see Fig. 3) [23]. This allows for greater flexibility across settings but the linearity of these models will ensure they still fail to capture how complex interventions are formed by their interaction with context

In retrospect, the initial logic model for the PET intervention was a type 2 logic model. Type 1 logic models are more basic than this, featuring a list of intervention components and outcomes, as popularised by the W.K. Kellogg Foundation [21]. These may be appropriate in the planning stage of an intervention’s lifecycle and have the benefit of being the least resource-intensive of logic model types but they do not describe aspects of context that are relevant to the intervention. The choice of a type 2 logic model over a type 1 logic model was therefore appropriate for the PET intervention because a central aim of the evaluation was to come to an understanding of the contextual factors which enable or constrain PET’s delivery. Yet, as we saw, the type 2 logic model could not model the complexity of the PET intervention. Its linear structure, proceeding from inputs to outputs/outcomes, meant that it could not convey how the intervention was being adapted through the action researchers’ facilitation. This is also the case with “system-based” logic models [22, 25] which describe implementation and context but assign them separate categories to the intervention, thus being an advanced form of type 2 logic model (Fig. 3).

It is common for researchers using type 2 logic models to recognise that their models poorly express intervention dynamics. Caveats can be included as to how logic models should be interpreted in the narrative that sits alongside any model (see Additional file 1: Appendix 1). For the PET intervention, the narrative would have to both explain the contents of the model and warn against a linear and rigid interpretation of it. Yet, this begs the question of whether alternative logic model types exist that could give a better elucidation of the PET’s dynamics. This would lessen reliance on the narrative and enable it to focus on explaining the core aspects of the intervention as captured in the model.

### Type 3 logic models

**Fig. 4:**
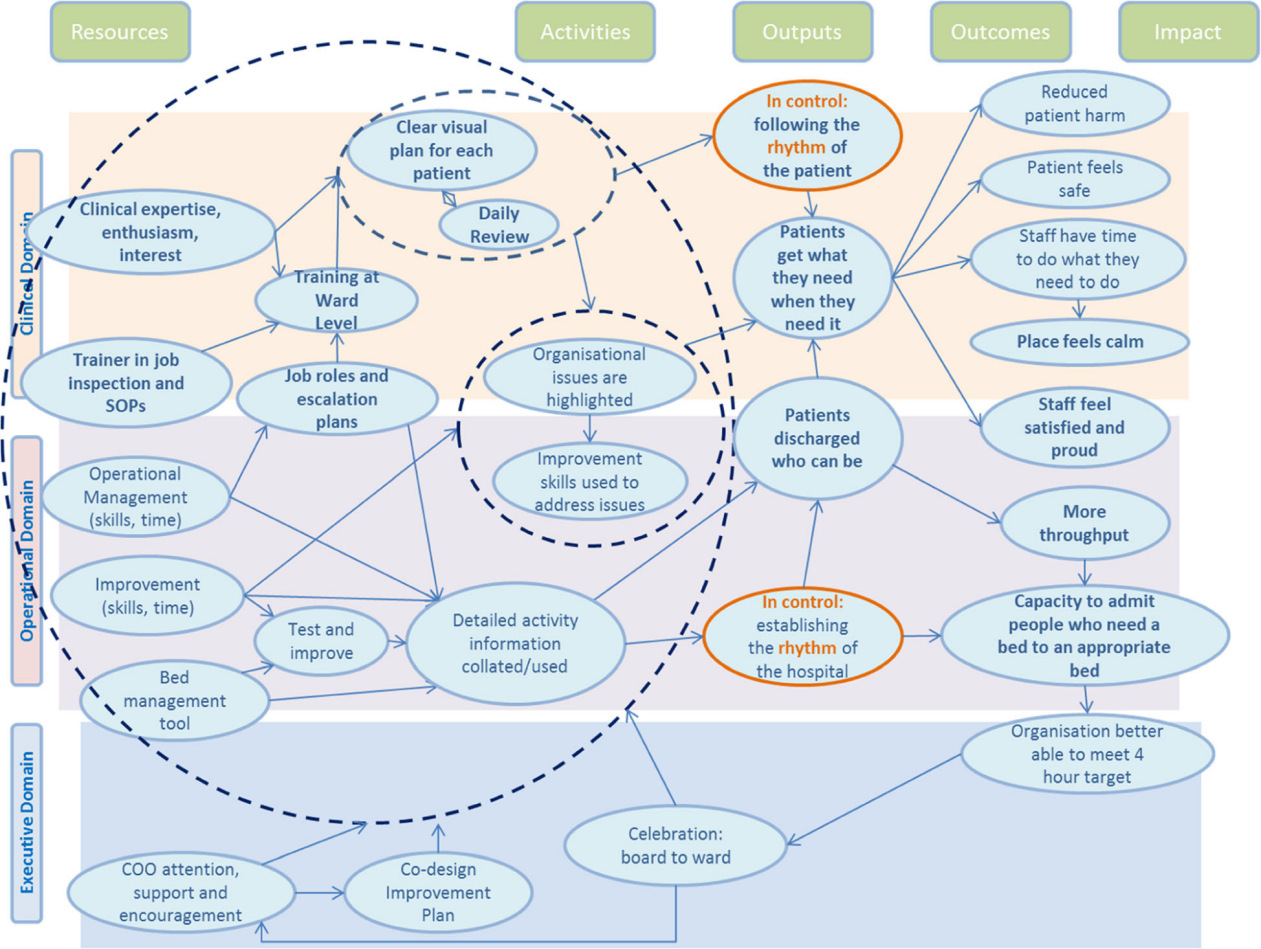
Example of a Type 3 Logic Model. This type 3 logic model expresses how a complex intervention works across multiple domains in a single setting, with interlinking actions producing a range of outputs and outcomes (Permission granted for publication by Beverley Slater, Improvement Academy Director)

Type 3 logic models draw connections between model factors and therefore more fully represent the logics of interventions, displaying exactly how they work to produce outcomes. A significant subset of these is “driver diagrams”, commonly used in improvement science [24]. They often include a precise list of intervention components and arrows that provide a clear sense of how each input leads to outcomes (Fig. 4).

These type 3 logic models can be useful to develop and test hypotheses related to the precise relationships between intervention components and outcomes. They are also often practitioner-oriented, used as part of consensus-building exercises about the requirement for change and how to go about it [24]. However, the focus of type 3 logic models is interventions rather than intervention settings and they are unable to accommodate interventions taking on a different form. Some type 3 logic models do incorporate “alternative causal strands”, enabling them to convey how interventions work in different settings [3, 12, 13]. Yet, the level of variation they can accommodate is limited to the number of strands they include. The question remains whether logic models can describe interventions which potentially take on a different form every time they are delivered.

### Type 4 logic models?

**Fig. 5:**
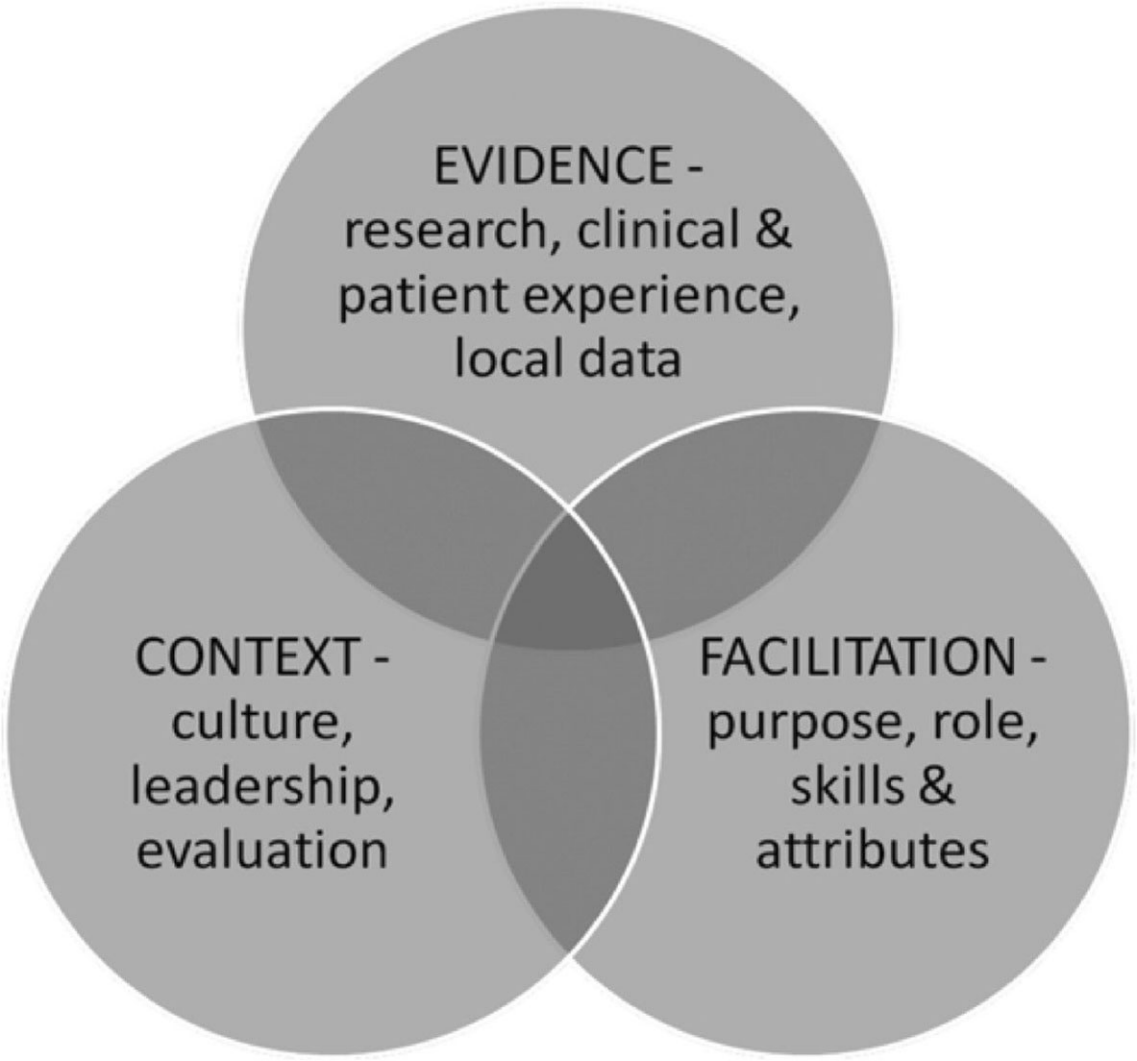
The PARIHS Framework. *Source: Hack* et al.*, 2011* [27]*.* PARIHS explains the success or failure of implementation projects in terms of an interplay between the evidence used in projects, the receptiveness of the context and whether the appropriate facilitation is provided. This is expressed using a three-way Venn diagram [14, 28–30]

An example of a model that successfully captures how the success of interventions hinges on their adaption to context is the Promoting Action on Research Implementation in Health Services (PARIHS) model (Fig. 5).

While the PARIHS model is not a logic model as such, the centrality it assigns to facilitation and context make it relevant to PET and indeed complex interventions in general which adapt on delivery through feedback loops [5]. In addition, while PARIHS has been used retrospectively to explain project outcomes, it can be used prospectively to plan implementation strategies before projects commence [14]. This is significant as it points to a potential new role for logic models of informing the development of context-sensitive facilitation strategies, as opposed to the traditional role of providing precise guidance as to how to act. In the next section, we incorporate aspects of the PARIHS model into a traditional logic model structure to create a type 4 logic model.

### Using PARIHS to model the PET intervention (Fig. 6)

Like PARIHS, our type 4 logic model aims to help future users of PET when they plan its implementation. It will be accompanied with guidance for them to prospectively assess contexts for its delivery and will include advice on how facilitators should respond to the moderators listed in the model, whether they are found to exert a positive or negative influence. Possible weaknesses include its high level of abstraction, which means that it does not provide precise guidance as to how facilitators should act but leaves it to them to decide when assessing the contexts in which they work. Additionally, because the model can accommodate the intervention taking on multiple forms across different setting, it places less emphasis on stakeholder agreement on model contents than traditional logic models. A type 4 logic model would therefore be inappropriate for use to establish agreement among stakeholders about the need for change and how to go about it.

**Fig. 6:**
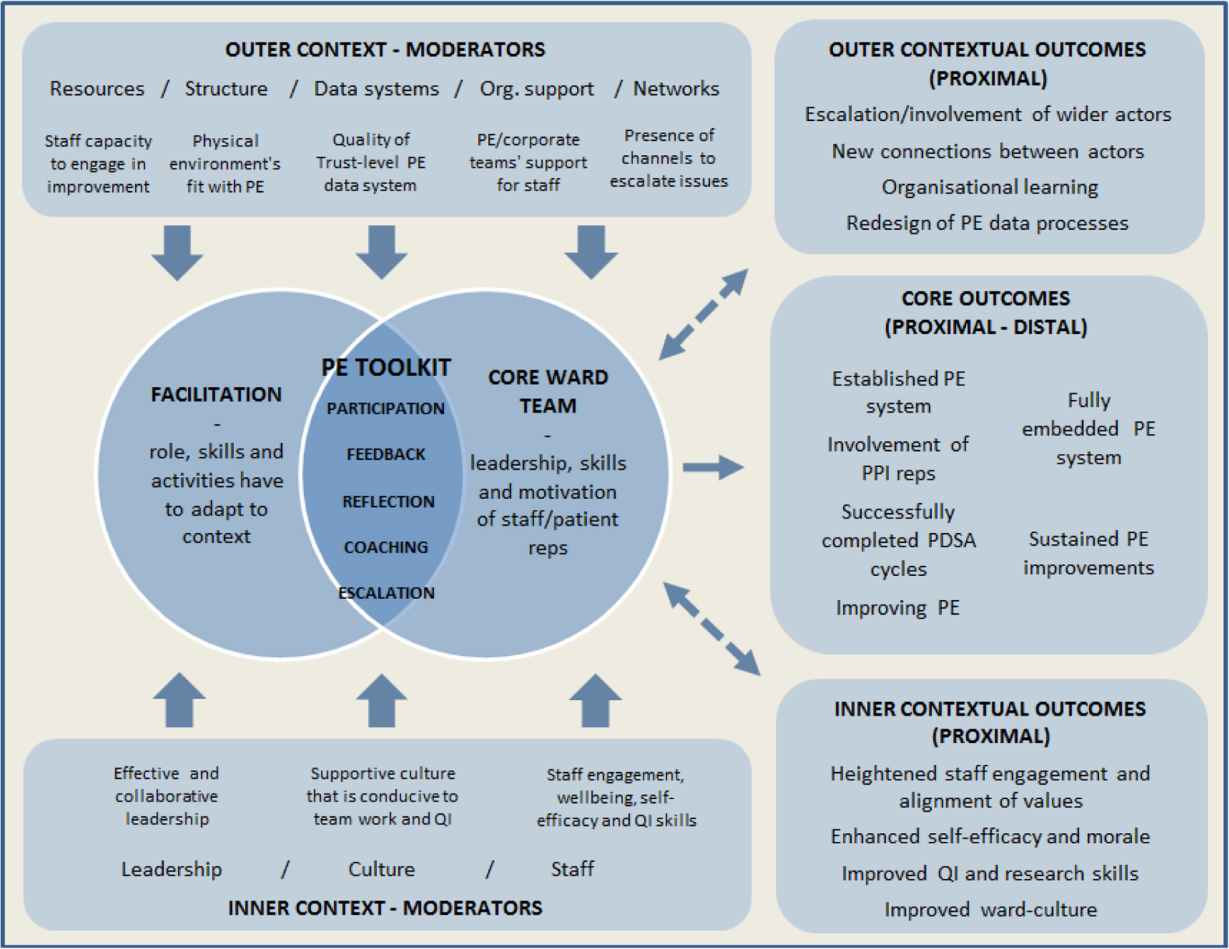
A Type 4 Logic Model for the PET Intervention. This type 4 logic models lists PET’s core intervention mechanisms (rather than a precise list of activities and resources) to allow for variation across settings while the model shape has been designed to address the inadequacies of the initial logic model identified above. 1. *Roles* – Where the initial model could not convey how the roles of facilitators and ward teams differed in each setting, the two circles of the Venn are designed to convey that roles and relationships must be adapted to fit the willingness and capacity of each ward team to engage. 2. *Interaction between the facilitation and moderators* – Although the PARIHS model assigns a single Venn circle to context, we have distinguished between contextual moderators that exert an influence from the outer context and the inner context. This is to show the full spectrum of factors that facilitators of PET must respond to for the intervention to succeed, either utilising positive moderators or overcoming negative moderators. 3. *Irregular patterns of proximal outcomes* – Core proximal and distal outcomes are listed to the centre-right, emerging if the intervention is successfully adapted to context. Context-dependent proximal outcomes linked to efforts to improve the receptiveness of ward settings to the intervention are situated at the top-right and bottom-right, in accordance with whether they target the outer or inner context respectively. The dotted arrows linking the Venn to these contextual, proximal outcomes convey the peripheral nature of these outcomes. 4. *Proximal outcomes influencing later success –* Finally, the double-headed arrows convey how the emergence of contextual, proximal outcomes can strengthen the work of the project

### Discussion: Principles for advancing the field of logic models

**Fig. 7:**
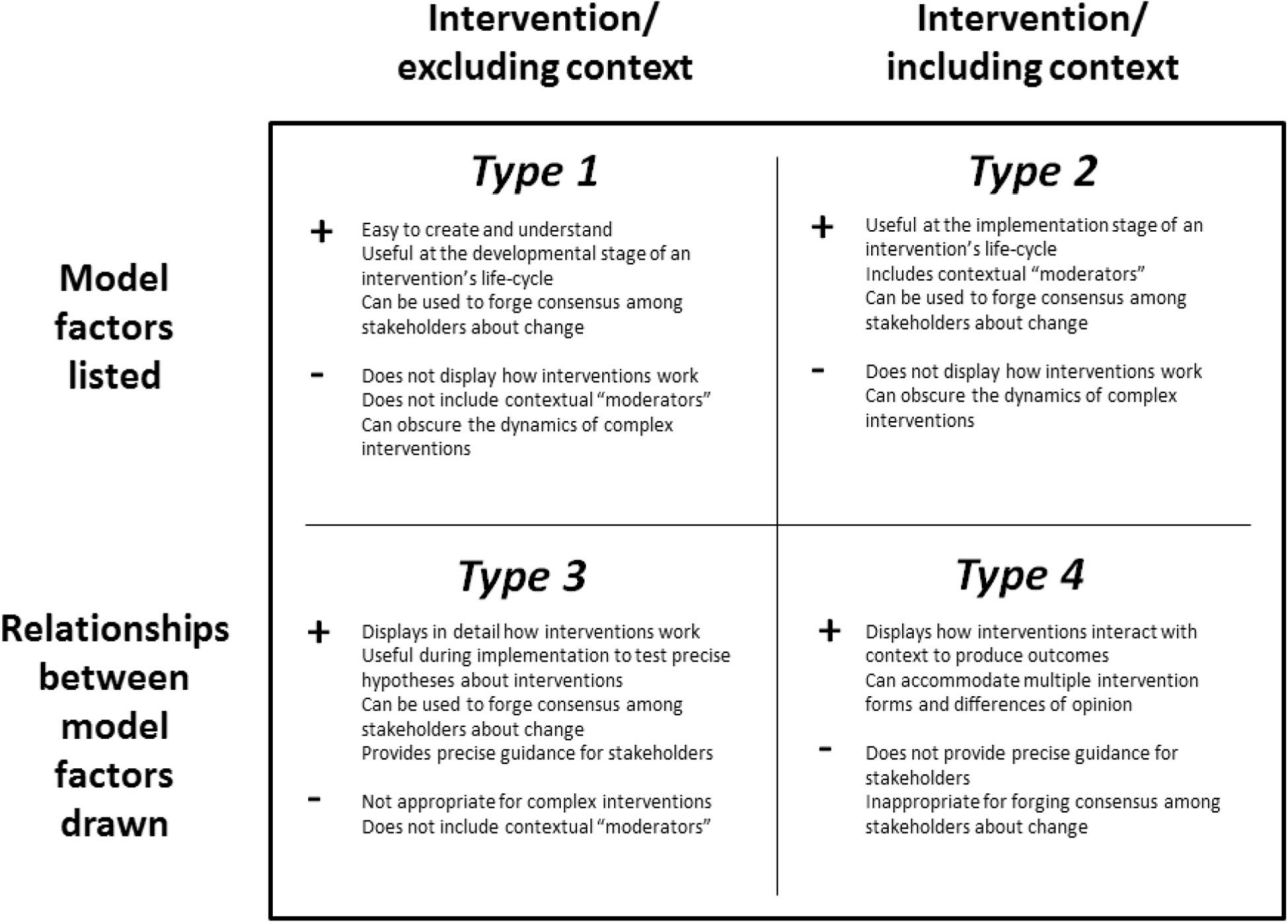
Strengths and Weaknesses of Logic Models Types. Researchers should be clear as to why they are using a logic model and experiment with different models to ensure they have the correct type

Our type 4 logic model approach shows that it is possible to qualitatively model the dynamics of complex interventions which potentially take on a different form each time they are delivered. However, it is important to recognise that type four logic models may not always be required. The “right” choice of logic model will be determined by the role it is to play in a given project and the complexity of the intervention at hand. If all that is required is a rough representation of an intervention and/or its delivery setting, type 1 or type 2 logic models will suffice. But if a fuller representation of intervention dynamics is necessary then a type 3 or type 4 model will be required. While Fig. 7 may help researchers to choose between different logic model types, here we draw upon our experience of developing a type 4 logic model to outline a formal methodology for how they may be derived.

#### Create logic models through robust qualitative research

To create a robust logic model, we recommend that researchers adopt a framework approach to qualitative data analysis [19] to manage and analyse data across multiple intervention sites. Logic model categories (intervention mechanisms, moderators and outcomes) can inform the columns of the framework matrix and each intervention site can be assigned a separate row, enabling potentially vast data to be organised and analysed so that model contents can be tested and refined in light of emergent categories and themes. This approach can be entirely inductive or combine deductive elements with prior theory informing the initial contents of the model. Testing against empirical data is crucial to ensure the robustness of the model.

In the case of interventions that are already known to be complex and adaptive, researchers can adopt an outline of our type 4 model and develop its contents in relation to the data contained within the framework matrix. Yet, it is likely that the level of complexity of an intervention will be unclear before it is tested, in which case researchers can experiment with different logic types as they are analysing their data. In our case, the PET intervention initially seemed *complicated*, with multiple component parts interacting in roughly similar ways [5]. Only by creating a type 2 logic model and testing and refining its contents did the full complexity of PET become apparent. We found that the type 2 logic model failed to convey 1) differences in the roles of facilitators and intervention users/recipients across settings 2) how the facilitators’ response to contextual moderators changed the shape of the intervention 3) irregular patterns of outcomes across different settings and 4) the influence of early proximal outcomes on the intervention’s later success. If interventions are found to share these characteristics, then a type 4 logic model will be necessary.

#### Use narrative to describe intervention logics

Narrative will always play a fundamental role describing the theoretical basis of interventions and explaining the content of logic models. If the narrative surrounding a model has to explain the inadequacy of a type 1, 2 or 3 type logic model to describe an intervention’s dynamics, this is a further sign that a type 4 logic model is necessary. In our case, we also listed the core intervention mechanisms in the model instead of a precise list of activities and resources to allow for greater variation in how interventions play out across different settings. This is consistent with a view of interventions as constituted by underlying mechanisms that are sensitive to context [31] or functions as opposed to precise forms, allowing for variation across different settings [2, 3]. The underpinning narrative should describe and reference the evidence-base for the mechanisms/functions, as is common in all logic model types [23].

#### Use diverse shapes and arrows to model dynamic relationships and contingencies

While one type 4 logic model shape has been proposed here, we encourage researchers to experiment with it to ensure a fit with their interventions. Wider policy analysis literatures highlight the potential of different types of lines and arrows to express dynamic relationships and contingencies in logic models, while it may be possible to use diverse shapes such as triangles and circles instead of a Venn diagram [3, 12, 13]. The key issue to remember, however, is that type 4 logic models must convey a dynamic relationship between the facilitation of an intervention, the users/recipients of the intervention, contextual moderators and outcomes. It is this level of dynamism which demarcates the approach from other logic model types. Revisions to our proposed type 4 logic model shape must therefore replicate how it displays the influence of context on intervention delivery and the functions of the Venn diagram and the dotted, double headed arrows in some form. The use of the Venn meant it was possible to model variation in terms of the roles and relationships of project facilitators and intervention recipients; the dotted, double headed arrows conveyed how certain proximal outcomes were contingent on the form the intervention took on and how they could improve the intervention’s functioning at a later stage.

#### Include the full spectrum of contextual moderators

Type 4 logic models are as much about context as they are interventions, consistent with the view of interventions as “events in systems” [2]. In our study, six diverse hospital wards/departments were involved, providing insight into the effects of context on the PET intervention. We drew upon frameworks of context to differentiate between moderators exerting influence from the “inner” and “outer” ward contexts while outcomes were categorised as “core” or “contextual”. An alternative would have been to use the micro/meso/macro distinction [32]. Either way, displaying the full spectrum of contextual moderators is vital to inform conversations about how interventions may be adapted to context or how and at what level the receptiveness of context may be improved.

#### Target logic models at facilitators

Because type 4 logic models are designed for complex, adaptive interventions which change shape across different settings, the traditional uses of logic models to forge consensus among stakeholders or provide precise guidance as to how to act to produce positive outcomes are increasingly irrelevant. However, because complex interventions adapt to contexts through a flexible facilitation function, making them “inextricably linked” to implementation and context [29], a new role for type 4 logic models emerges: to guide how future users of complex interventions adapt them to context. While all logic models are accompanied with a narrative of some sort, in the case of type 4 logic models this can be tailored to inform facilitators’ assessments of context and to enable them to develop context-sensitive facilitation strategies. This may enhance the scale-up of complex interventions.

#### Incorporate differences of opinion

Finally, while we recognise that the type 4 logic models we propose will be less suitable for forging agreement among stakeholders than traditional logic models, accommodating differences of opinion may be more suitable for complex interventions given that the potential for disagreement increases with more complex problems [33]. Here, it is interesting to note that some report logic models to have caused unnecessary friction when used to forge consensus over a proposed change [15] while others have warned they supress marginalised voices [12, 16]. In our case, stakeholders had different views on the PET document, with some viewing it as central to the intervention and others peripheral. Stakeholders also disagreed on the order of significance of the moderating factors: some downplayed the significance of staffing pressures while others argued that improvement work was not possible without addressing these first. Rather than resolve these differences or prioritise one over the other, our model allows for the possibility that both are right in different settings.

## Conclusion

In this paper, we have proposed a typology of logic models, including strengths and weaknesses, to help researchers select between different logic model types in intervention research. In addition, we have outlined a formal methodology for developing more dynamic logic models than those which currently exist, incorporating aspects of the PARIHS model into a traditional logic model structure. These “type 4” logic models are capable of expressing interaction between interventions and context but some change to how logic models are used is required. Because type 4 logic models are designed for complex interventions which change shape across different settings, the traditional uses of logic models of forging consensus among diverse stakeholders and/or providing precise guidance as to how to act to produce positive outcomes are increasingly irrelevant. We propose that type 4 logic models should be developed and refined through rigorous qualitative research rather than consensus-building exercises. In addition, they should seek to guide future users of complex interventions to help them develop context-sensitive facilitation strategies. A benefit of this approach is that it may enhance the scale-up of complex interventions.

## Additional file

Additional file 1: Appendix 1. Glossary of key terms of logic models. (DOCX 15 kb)

## Abbreviations

BMC: BioMed Central
MRC: Medical Research Council
PARIHS: Promoting Action on Research Implementation in Health Services
PET: Patient Experience Toolkit

## Footnotes

1 HS&DR commissioned the Yorkshire Quality and Safety Group of the Bradford Institute of Health Research to carry out the study
