# Supplementary material for: Advancing complexity science in healthcare research: the logic of logic models
Source: BMC Med Res Methodol. 2019 Mar 12;19:55. doi: 10.1186/s12874-019-0701-4 (PMC6419426; doi:10.1186/s12874-019-0701-4)
Supplement: Supplementary file 1 — Appendix 1. Glossary of key terms of logic models. (DOCX 15 kb) [file 12874_2019_701_MOESM1_ESM.docx]

# Additional file 1: Appendix 1

**Glossary of key terms of logic models**

*Theory* – Theory can refer to the overarching programme theory of interventions and the potentially numerous hypotheses, or “If this, then this” statements, which constitute programme theory and form the basis of logic models. A role is recognised in the literature for both formal, scientific theory and informal theory based on tacit assumptions and observations in developing hypotheses about how interventions work [23]. The term “mid-range” theory, designed to differentiate against grand social theories (such as psychoanalysis or Marxism), refers to formal theories that may be relevant to an intervention (such as diffusion of innovations theory) or evidence on some core intervention mechanism (such as team working or reflection) [23].

*Narrative –* Programme theory can be presented in narrative or diagrammatical form. A narrative can be used instead of a logic model, where the programme theory can be presented in a series of hypotheses in the form of “if, this, then, this” statements. Or narrative can be as a complement to a logic model, explaining what the logic model does and providing what Davidoff et al call a “narrative account” of an intervention’s theoretical basis, including its rationale, assumptions and links to wider theoretical literature [23]. Most if not all logic models are supplemented with a detailed description of model elements, allowing additional detail to be conveyed without overcrowding the models [22].

*Inputs/resources* – Numerous terms can be used to refer to the core components of interventions. Inputs and resources refer to the raw materials of interventions, which can include anything from the knowledge and skills of practitioners to a clinical protocol or toolkit for implementation.

*Activities/mechanisms/drivers* – Activities refers to the actions and processes through which inputs/resources are utilised by stakeholders to create change, while mechanisms similarly refers to the way in which resources prompt a response from stakeholders [33]. The term activities directs attention to precisely how an intervention takes shape whereas mechanisms is more abstract, often referring to underlying factors and processes that cause outcomes. Drivers is a term often used in Quality Improvement and shares similarities with mechanisms [24].

Context/moderators – Context is a crucial determinant of whether interventions succeed in improvement and implementation projects [31]. Context includes anything that exists in a setting prior to delivery of an intervention, although intervention resources can include aspects of context, making it difficult to differentiate between the two [33]. Moderating factors present in a context enable or constrain interventions and can be thought of as mechanisms in their own right; identifying them requires developing theory about what aspects of context shape interventions.

*Outputs/mediators* – Outputs/mediators refer to the immediate product of intervention activities and are the link between interventions and outcomes. Examples include changes to participants’ behaviour, knowledge and level of functioning which pave the way for a service improvement.

*Outcomes (proximal/distal)* – An alternative term for outputs/mediators is proximal or short-term outcomes, while distal outcomes refers to the ultimate goals that interventions are designed to achieve.
